# On the Improvement of Free-Energy Calculation from Steered Molecular Dynamics Simulations Using Adaptive Stochastic Perturbation Protocols

**DOI:** 10.1371/journal.pone.0101810

**Published:** 2014-09-18

**Authors:** Ognjen Perišić, Hui Lu

**Affiliations:** 1 Shanghai Institute of Medical Genetics, Shanghai Children’s Hospital, Shanghai Jiaotong University, Shanghai, China; 2 Department of Bioengineering, University of Illinois at Chicago, Chicago, Illinois, United States of America; University of Leeds, United Kingdom

## Abstract

The potential of mean force (PMF) calculation in single molecule manipulation experiments performed via the steered molecular dynamics (SMD) technique is a computationally very demanding task because the analyzed system has to be perturbed very slowly to be kept close to equilibrium. Faster perturbations, far from equilibrium, increase dissipation and move the average work away from the underlying free energy profile, and thus introduce a bias into the PMF estimate. The Jarzynski equality offers a way to overcome the bias problem by being able to produce an exact estimate of the free energy difference, regardless of the perturbation regime. However, with a limited number of samples and high dissipation the Jarzynski equality also introduces a bias. In our previous work, based on the Brownian motion formalism, we introduced three stochastic perturbation protocols aimed at improving the PMF calculation with the Jarzynski equality in single molecule manipulation experiments and analogous computer simulations. This paper describes the PMF reconstruction results based on full-atom molecular dynamics simulations, obtained with those three protocols. We also want to show that the protocols are applicable with the second-order cumulant expansion formula. Our protocols offer a very noticeable improvement over the simple constant velocity pulling. They are able to produce an acceptable estimate of PMF with a significantly reduced bias, even with very fast perturbation regimes. Therefore, the protocols can be adopted as practical and efficient tools for the analysis of mechanical properties of biological molecules.

## Introduction

Proteins with mechanical functions can be roughly divided into two large groups. Members of the first group are mechanically active which means that they perform biological tasks by generating a mechanical force through their own conformational changes. Such a force is usually observed during a stretching experiment as an increased mechanical resistance to pulling. Spectrin, a protein that forms the cytoskeleton in red blood cells and muscle protein titin are examples of proteins with a direct mechanical role. Members of the second group are force sensitive proteins. They respond to a mechanical stimulus by changing their overall three dimensional structure. Such changes trigger cascades of various biochemical processes through the interactions these proteins have with other biomolecules. The force sensitive proteins thus serve as signal transmitters and signal transducers. Examples of mechano-sensitive proteins are integrins, cadherins, fibronectin, and bacterial adhesive protein *fimH*.

Mechanical properties of proteins belonging to either of these two groups are determined by their potentials of mean force (PMF), i.e., free-energy profiles along the reaction path. The knowledge of PMF is therefore crucial for the complete understanding of the functioning of a protein with a mechanical role. The real-world single molecule manipulation with the atomic force microscopy (AFM) [1], [2], [3], [4] or with the optical tweezers [5], and the simulated stretching via the steered molecular dynamics (SMD) technique [2], [3], [4], [6], [7], [8] can provide data for the PMF reconstruction. Simulation methods, although easy to perform due to advances in software and hardware, often cannot produce enough data for a good quality PMF reconstruction. Their limitations stem from the numerical complexity of simulation protocols, which require fast, and therefore, far from equilibrium perturbations. A fast perturbation inevitably increases dissipation and a corresponding PMF estimate. Advances in nonequilibrium statistical mechanics, especially the introduction of the Jarzynski equality [9] opened a way toward a more accurate extraction of equilibrium properties from nonequilibrium experiments. However, the slow convergence of the Jarzynski equality with the increase in the number of samples [10] seems to limit its applicability to near equilibrium perturbation regimes only.

The potential of mean force may be perceived as a one-dimensional function of a reaction coordinate, but it is, in fact, a multidimensional free-energy profile averaged over all degrees of freedom, except the very reaction coordinate being examined. That multidimensionality means that a pulled protein may follow very different unfolding pathways during repeated experiments and thus may exhibit irreversible behavior during a short duration simulation (or experiment). A protein accidentally unfolded by a random fluctuation may require more time to refold to the initial state than available during a short duration SMD stretching simulation (on protein folding times see Ref. [11]). Furthermore, protein unfolding (or protein-ligand unbinding) is a process in which the friction coefficient is a function of the reaction coordinate. The friction between a polymer and its surrounding determines the amplitude of thermal fluctuations. Brownian motion simulations are usually performed with fixed diffusion/friction coefficients, which means that the variance of their fluctuations is constant. In real-world situations, protein structural elements, open loops for example, often have very slow relaxation times, and make the friction and the very stochastic behavior of polymers dispersive [12].

The aim of this paper is to present three stochastic perturbation protocols we developed in attempt to improve the PMF calculation with the Jarzynski equality. The protocols were introduced in our previous publication [13] in which we used a simple one-dimensional Brownian motion model to simulate the fast polymer stretching. Brownian simulations are useful due to the easiness of their implementation [14], [12], [15], and the speed with which they can be performed, but they are only crude simplifications of real-life processes. Here we present results of our research based on full-atom molecular dynamics simulations of a deca-alanin stretching (deca-alanin is a peptide made of ten alanin amino-acid residues).

The paper starts with a short overview of the theory of free-energy calculation. Simulation and reconstruction procedures are described in the second chapter. In that chapter we also introduce the weighted histogram formula [14], used to implement the Jarzynski equality, together with the second order cumulant expansion formula [16]. In the next chapter, we describe a standard SMD constant velocity perturbation protocol, analogous to the protocols applied with real-life protein manipulation tools, such as the atomic force microscopy (AFM), or the optical tweezers. In that chapter, we also deal with the maximum bias estimation. After that, we present results based on a simple stochastic perturbation protocol, which offers only a limited improvement over the normal constant velocity pulling. After that, we introduce two improvements of the stochastic protocol. Finally, we compare the efficiency of the three protocols. The paper ends with the Conclusion.

## Theory

All systems in nature dissipate energy when they perform work, or when the external work is performed on them. That means that the average external work used to perturb a given system between two states is usually larger than the corresponding free-energy difference, 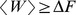, a fact described by the Second Law of Thermodynamics [17]. The mean work is usually larger than the free energy difference because a part of the external work is always dissipated. The equality is satisfied only if the external perturbation is reversible, i.e., if it is infinitely slow. However, an infinitely slow perturbation is difficult to perform or simulate which means that practical perturbations are usually non-equilibrium processes.

Various theoretical, numerical and perturbation methods were developed in attempt to improve the calculation of free energy. Those methods move a given molecular system between two terminal states by means of an external potential. Unfortunately, they can produce an accurate estimate of Δ*F* only if the perturbation is performed very slowly. Real-life, non-reversible perturbations introduce a bias into the estimate. That bias can be reduced through a further reduction of the perturbation rate, which may be computationally very expensive.

In 1997, C. Jarzynski presented a theoretical framework able to directly connect the exponential average of the external work performed during the perturbation and the corresponding free-energy difference [9]

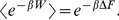
(1)


The most important property of this relation is that it is not restricted to close to equilibrium regimes only. The equality is satisfied for any perturbation, only if enough samples are available [10].

Theoretically speaking, the Jarzynski work relation is equality, but in practice, with a limited number of work samples, it generates a noticeable bias [10], [16]. It was shown that for a bias larger than 15 *k_B_T*, and equilibrated heath bath [18], the number of samples required to reduce that bias to an acceptable level is larger than a number of samples anyone can expect to obtain experimentally [10]. Such a bias is noticeable with fast pulling steered molecular dynamics (SMD) experiments [6], [16]. A fast SMD perturbation, at least three orders of magnitude faster than real-word AFM experiments, generates a significant dissipation which may raise the Jarzynski bias to a level which can hardly be reduced through the increase in the number of samples [10], [16].

Our research aim was to develop a new perturbation protocol able to produce a good quality PMF reconstruction with a limited number of samples. We wanted to enable the accurate calculation of free energy using the fast pulling steered molecular dynamics technique. The initial theoretical work on this problem was presented in our previous publication [13] in which we used a simple, Brownian motion model to develop three stochastic pulling protocols. Here we present a full-atom SMD implementations of the same three protocols.

The method development requires many repeated trials, which means that the target system has to be made of a small number of atoms. The small number of atoms allows fast computation and multiple SMD simulations. Deca-alanine, a peptide made of ten alanine residues (104 atoms) is ideal for that task because it keeps a helical structure (2 whole helical turns) in vacuum, on a room temperature [19]. That means that its pulling can be carried out without a solvent. The solvent elimination reduces the computational costs, but requires the application of the Langevin dynamics to control the system’s temperature and introduce random fluctuations otherwise caused by a thermal environment. Deca-alanin was already used to test the nonequilibrium work relation [16], [20]. The results based on the constant velocity pulling showed that the Jarzynski equality can give a relatively accurate PMF estimate, with a limited number of samples, only in a close to equilibrium regime. That regime for deca-alanin is achieved when the pulling velocity is 1 m/s or slower. A ten times faster pulling (10 m/s) introduces a bias which even 10,000 puling trajectories cannot reduce [16].

## Simulation and reconstruction procedures

In a SMD simulation, a biological polymer is stretched analogously to the stretching performed with AFM [1], [2], [3], [4] or with the optical tweezers [5]. In that kind of experiment, one end (atom) of a molecule is fixed, and the other end is moved by a cantilever. The cantilever, usually interpreted as a Hookean spring, connects the terminal end of a pulled molecule (henceforth pulled point) to the external point (pulling point, from now on) which moves with the predetermined protocol. In a simulation, that cantilever is virtual, i.e., it does not occupy any space, but influences the pulled point as an existing entity.

Two perturbation protocols are dominant in the polymer stretching; one is the constant velocity pulling (normal pulling) and other is the constant force pulling. Both of them can be used to generate data for a PMF calculation. In this work, we are focused on the constant velocity pertrubation.

The Jarzynski equality (Eq. 1) cannot be directly used to calculate PMF because it only gives the free-energy difference between two states, whereas PMF represents the evolution of free-energy along the reaction coordinate. Hummer and Szabo [14] adapted the Jarzynski equality to PMF calculation using the *weighted histogram method* [21]

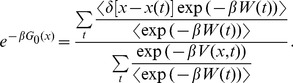
(2)The term *G*_0_(*x*) in this formula is the value of the Gibbs free-energy at a specific point *x* along the reaction path, *W*(*t*) is the work sample at time *t* and *V*(*x*,*t*) is the external potential (instantaneous deflection potential). We will use this equation for the Jarzynski free-energy calculations.

The *second-order cumulant expansion* formula is used to approximate the Jarzynski equality [16], [22], [23]. The cumulants are obtained by expanding the logarithm of a characteristic function. The second-order approximation is based on the external work distribution properties in equilibrium. If the external work has a normal distribution than all cumulants after the second one are identical to zero. Only in that case, the formula for the free-energy calculation is, without loss of generality,
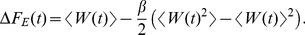
(3)


This relation is identical to the near equilibrium formula [24], [25]. Park et al. [16] showed that the *stiff spring approximation* [12] forces a polymer to adopt the quasi equilibrium behavior, characterized by a normal distribution of the external work, thus allowing the application of Eq. 3 without limitations. The stiff spring imposes a short relaxation time, which allows the perturbed system to maintain the equilibrium-like behavior. This approximation also interprets the external work used to pull the polymer-spring system as a work used to stretch the polymer only. The approximation assumes that the increased stiffness keeps the pulling spring short, thus making the instantaneous deflection potential negligible. The second order cumulant expansion shows much faster convergence with biopolymers [16]. We did not use the third-order cumulant expansion formula because it produces rather noisy PMF estimates [26]. We will show later that the stiff spring approximation is not always applicable, and that the external work can deviate from the Gaussian distribution.

We used the NAMD simulation package [27] with the CHARMM22 force field [28] to simulate the deca-alanin stretching. The initial helical configuration was obtained from the web site of Klaus Schulten’s *Theoretical and Computational Biophysics group* (http://www.ks.uiuc.edu/Training/Tutorials/). The Langevin dynamics was applied with the damping coefficient *γ*  = 5 *ps* ^−1^ and the pulling spring stiffness was 500 pN/Å. The perturbation protocols were implemented in the *Tcl/Tk* script language embedded in the NAMD package. The simulation package allows the application of a 2 fs time step via the SHAKE integration algorithm. We used the same simulation parameters and the same pulling velocities (1 m/s and 10 m/s) as Park et al. used [16]. The first pulling velocity, 1 m/s, corresponds to the close to equilibrium perturbation and produces a very good estimate of PMF, whereas ten times faster velocity, 10 m/s, introduces a significant bias. We, therefore, applied the stochastic perturbation protocols to that velocity only. With each simulation setup, we generated 10,000 trajectories, except for the 1 m/s pulling, with which we generated 1000 trajectories only. Besides using all 10,000 trajectories per simulation setup to calculate PMF, we used smaller subsets of 100, 200, 500, 1000, 2000, 4000 and 8000 trajectories also. When the number of trajectories per subset allowed multiple reconstructions, we calculated all possible PMF estimates using separate non-overlapping subsets (for example, 100 reconstructions based on 100 trajectories, and so forth). We applied the two aforementioned reconstruction procedures, (Eqs. 2 and 3) to calculate deca-alanin's PMF at 500 points along the 20 Å long reaction path.

### Referent and benchmark PMF estimates

To estimate a referent deca-alanin’s PMF, we generated 40 trajectories using the normal pulling protocol with a very slow pulling velocity, 0.002 m/s, and applied Eq. 2 on them. Those 40 slow pulling trajectories are computationally as expensive as 200,000 trajectories generated with the 10 m/s velocity. To estimate the accuracy of that prediction, we used a bias estimating procedure, based on the work of Gore et al. [10], that uses the estimate’s fluctuations to establish the distance of that estimate from a true free energy profile (the detailed description of that procedure is given in the Chapter 3). The maximum bias our 0.002 m/s Jarzynski PMF estimate, thus established, is less than 1 *k_B_T*.

We also calculated a set of PMF estimates using the normal constant velocity pulling protocol, with two velocities, 1 m/s and 10 m/s. We used those estimates as benchmarks.

## Constant velocity pulling and the maximum bias estimation

**Figure 1:**
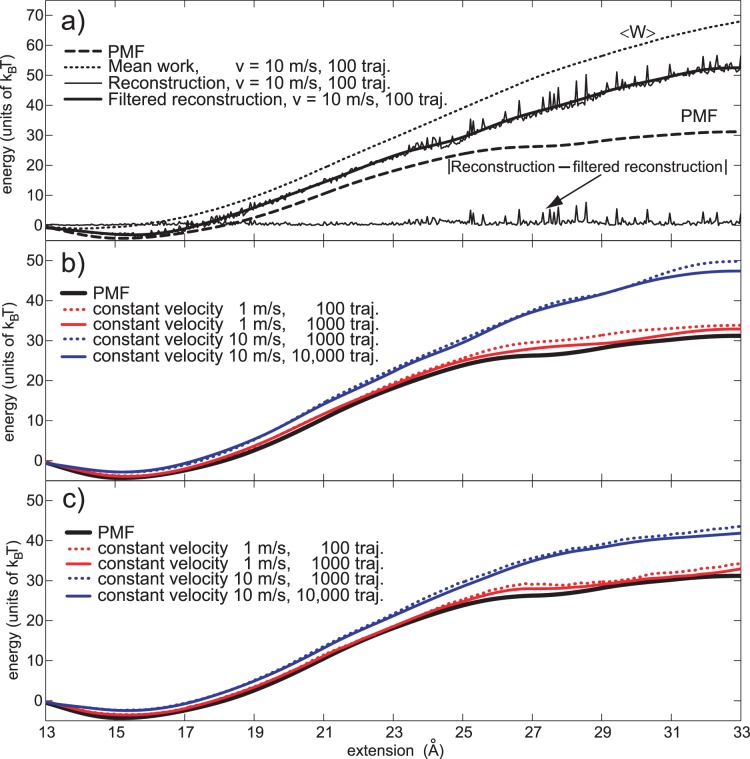
Normal pulling based estimates a) Jarzynski based PMF estimate based on the normal 10 m/s pulling and limited number of trajectories (100). The thin and “noisy” line is the estimate, and the thick line passing through it is its smoothed version. The line on the bottom is the absolute difference between the estimate and its smoothed version; it represents the estimate’s fluctuations. We used these fluctuations to analyze the behavior and determine the maximum bias of our estimates. The dashed line is PMF. The dotted line is the mean work. b) PMF estimates based on the normal pulling protocol and the Jarzynski equality, for 1 m/s and 10 m/s pulling velocities. c) PMF estimates based on the normal pulling protocol and the cumulant expansion formula, for 1 m/s and 10 m/s pulling velocities. For each pulling velocity two estimates are given, one based on the maximum number of work trajectories, and the other on 10 times less trajectories. With the slower pulling, we used fewer trajectories in order to make the comparison on equal terms, i.e., using the same computational cost.

A Jarzynski PMF estimate based on a limited number of trajectories is not very smooth, it contains fluctuations [10], [13]. An example of those fluctuations can be seen in Fig. 1a. When the number of work samples is small, it can be assumed that the estimate’s fluctuations are not an intrinsic property of the potential but a byproduct of dissipation and limited sampling [10], [13]. The potential of mean force of a biological molecule cannot contain high frequency fluctuations along the reaction path because such fluctuations imply high amplitude forces that are (presumably) not possible with biological polymers. The high frequency changes of PMF along the reaction path would produce forces, as first spatial derivatives of the potential, much higher than the forces encountered in natural biomolecular systems.

The easiest way to extract the fluctuations from a Jarzynski PMF estimate is to apply a digital filter to it. The knowledge of the estimate’s harmonic spectrum is essential for that operation. The analysis of the harmonic spectrum of an average deca-alanin Jarzynski PMF estimate using the Fourier spectral analysis [29] shows that only low frequency harmonics (the ones covering up to 4% of the sampling rate) have a significant contribution to its shape. The rest of the spectrum has two orders of magnitude smaller amplitudes and probably belongs to the fluctuations caused by the limited sampling. Knowing that, we applied a low-pass filter (Butterworth filter) [30] with a 20 dB stop-band attenuation and a sinusoidal cutoff frequency which is 3% of the sampling frequency (for 500 sample points along the reaction path). We used the numerical package MATLAB to perform the filtering. The second-order cumulant expansion formula (Eq. 11) does not require filtering because it produces very smooth estimates. The second order expansion is itself a sort of filtering procedure because it disregards the tails of the work distribution and uses only the slowly changing variance, besides the mean value of the external work.

Fig. 1b shows smoothed Jarzynski estimates and Fig. 1c second-order cumulant-expansion reconstructions. These two figures show that the faster pulling produces much better results with the cumulant approximation than with the Jarzynski equality; the cumulant-expansion formula bias at the end of the reaction path is noticeably smaller than the analogous Jarzynski bias. The slow pulling produces the same quality of reconstruction with both averaging schemes because its work distribution is very narrow, with negligible tails. Those tails are responsible for the higher bias of the Jarzynski estimates when pulling is faster. It is also obvious that the bias falls very slowly with the increase in the number of samples (ten times more samples is much less effective than ten times slower pulling [16]). However, this conclusion is applicable to this case only (a small molecule and narrow-width thermal fluctuations).

Fluctuations of a PMF estimate are not desirable because they represent an inaccuracy, but they can be useful because they carry information on the amount of bias. Gore et al. [10], as well as Zuckerman and Woolf [31], [32] connected the fluctuations of a Jarzynski estimate *σ_J_*(*N*) to its bias *B_J_*(*N*) when the number of samples *N* is large and the heat bath is equilibrated (Eq. 9 in ref. [10]):
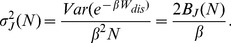
(4)


We used this formula to calculate the maximum bias of our Jarzynski PMF estimates based on the 10 m/s normal pulling (and to calculate the bias of the referent 0.002 m/s velocity based estimate). The thin line on the bottom of Fig. 1a shows the estimate fluctuations obtained by subtracting the smoothed estimate from the original one. That line clearly shows that the amplitude of the fluctuations rises with the bias. Those fluctuations were obtained by subtracting the filtered reconstruction from the original PMF reconstruction. They were used together with Eq. 4 to obtain the estimate of the maximum bias. Multiple experiments showed that this method produces the best results when the mean value of the estimate’s fluctuations plus one standard deviation are taken as the fluctuations maximum amplitude. This approach is valid only if the underlying PMF is a slowly changing function.

**Figure 2:**
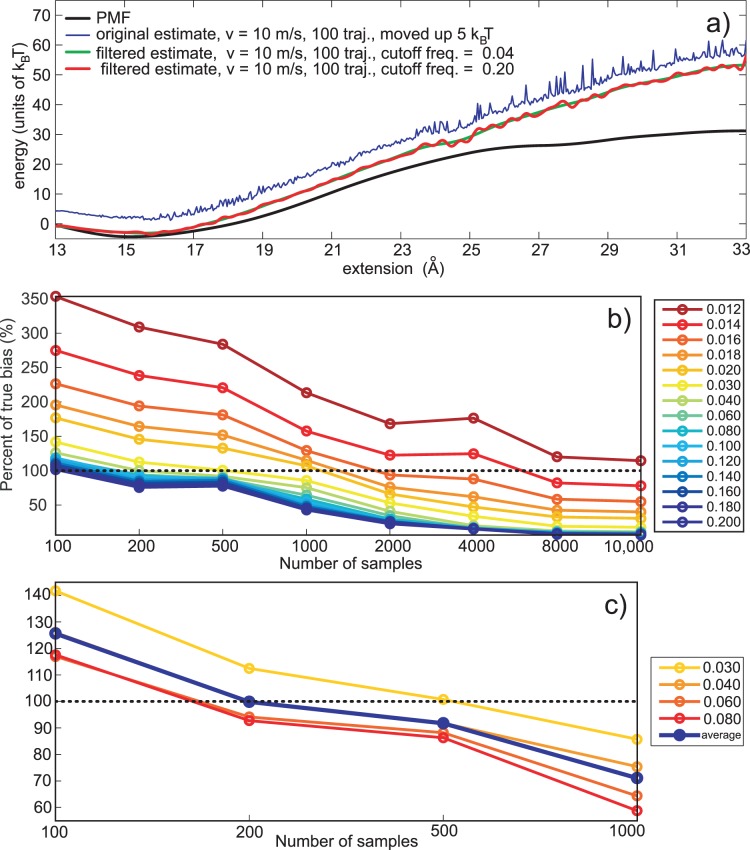
Maximum bias estimation. a) Original PMF estimate (blue line, moved up by 5 *K_B_T* for a clearer picture) in comparison to its two filtered (smoothed) versions. The first version (the green one) is produced by applying a low pass filter (Butterworth) with a very low cutoff frequency (2% of the sampling frequency). The second filtered output (the red one) is also produced by the low pass filtering, but with a much higher cutoff frequency (20% of the sampling frequency). b) Quality of the bias estimating based on the low-pass filtering technique. The quality is expressed as the percent of the true bias predicted for a given number of pulling trajectories. The cutoff frequencies are in the range 0.012 to 0.20 (1.2 to 20%) of the sampling frequency. The dashed line designates the actual bias for a given number of trajectories. c) Maximum bias predictor behavior for a limited number of work trajectories per reconstruction (100 to 1000). The behavior of the maximum bias predictor (red line), as an average of four predictors based on 4 different cutoff frequencies, (0.03, 0.04, 0.06 and 0.08, blue lines).

We used the low-pass Butterworth digital filter to obtain the estimates’ fluctuations and we tested that approach with a whole range of filtering frequencies, from low cut-off values, which pass through the filter just a few harmonics, to much higher cut-off values, whose outputs accurately follow the shape of a Jarzynski PMF estimate. If the cutoff frequency were identical to the sampling frequency, the filter would not remove anything from the input signal. In that case, the filter’s output would be the original input signal (a Jarzynski PMF estimate). As an example of the filtering behavior, see Fig. 2a. The red line in that figure minutely follows the original PMF estimate (thin blue line), while the green line exhibits much less fluctuations – it is more stable. The red and green lines are smoothed PMF estimates obtained by filtering the original PMF estimate using two different cut-off frequencies. The green estimate was produced with a very low cutoff frequency, and the red one with a ten times higher cutoff. The subtraction of the red line from the original, blue PMF estimate produces fluctuations that are in a good correlation to the bias. The green line, on the other hand, overestimates the bias because it does not accurately follow the fluctuations, it goes straight through the estimate, and thus overestimates the fluctuations amplitude(s). The green line is very smooth and mostly misses the local behavior of the original estimate. That implies that the red line should be chosen to extract the fluctuations. Therefore, without the knowledge of the underlying bias, using just an observation of the output of the described filtering procedure, one can (roughly) estimate the correct frequency (or frequency range) to be applied in the Jarzynski bias estimation.

To test this assumption we applied the filtering procedure with various cutoff values, starting with 1.2% and going to 20% of the sampling frequency. The behavior of bias estimates based on these frequencies is depicted in Fig. 2b. The quality of the bias prediction is expressed as the ratio of the bias prediction versus the true bias for a given number of samples and a given cutoff frequency. Every point is the average of multiple experiments (100 bias estimates for 100 trajectories per reconstruction, and so on). The figure shows that the best bias estimates for a small number of trajectories per reconstructions (100 to 1000) are obtained with higher cut-off values (12% to 20%, i.e. 0.12 to 0.20). In those cases, bias estimates are between 60% and 130% of the true Jarzynski bias. Cutoff values lower than these (1.2% to 6%) hugely overestimate the bias because the filtered output they produce is not able to follow the overall shape of the original Jarzynski estimate. That happens because the filtering procedure passes through only a few sinusoidal harmonics that are not able to accurately describe the input signal. The output is either above or below the input. The fluctuations amplitude thus obtained is very high and overestimates the bias when applied via Eq. 4.

A large number of trajectories per reconstruction (2000 or more in this case) produces Jarzynski estimates with small amplitude, long wavelength (low frequency) fluctuations that cannot be extracted with the above described high frequency filtering. The filtering based on a high cutoff frequency cannot see those low amplitude fluctuations, because it is local in nature; it only sees a very narrow region around the reconstruction point (see Fig. 2a), for that matter it is similar to the moving average procedure. Long wavelength fluctuations can be extracted using only very low cutoff frequencies, lower than 2% of the sampling rate. The upper curve in Fig. 2b, (red line) which corresponds to the 0.012 cutoff frequency shows that bias estimates for this frequency, based on 8000 and 10,000 trajectories are relatively accurate, as opposed to estimates based on a smaller number of trajectories. Therefore, with many work samples, the cutoff frequency should be low. However, a large number of work trajectories is very expensive to produce, and not necessary, because the average Jarzynski bias falls slowly with the increase in the number of samples (see Fig. 1b and Ref. [10]).

We gave an overview of the proper way to design the filtering procedure, but the cutoff frequency can be estimated through a simple observation of the filtered output. It should follow the overall shape of a PMF estimate, but it should follow its local behavior as well.

To produce a robust predictor of the Jarzynski bias, for a small number of samples, we averaged the output of four best bias predictors based on four cutoff frequencies (0.03, 0.04, 0.06 and 0.08). Those frequencies produce the best predictions with estimates based on a small number of samples per reconstructions (100 to 1000). Fig. 2c depicts the behavior of the averaging bias predictor (red line), and four predictors based on the individual cutoff frequencies, for the reconstructions based on 100, 200, 500 and 1000 trajectories. It is clear that with 200 samples per reconstructions the averaging predictor accurately estimates the bias. With 100 samples only, the procedure overestimates the bias. We will show later that a moderate bias overestimate (less than 30% in this case) is not a drawback when that bias prediction is used to estimate the amount of the external noise required to improve the external work sampling.

Figure 2c shows that the filtering of PMF estimates based on a small number of work trajectories (100–1000) using medium to high frequencies produces acceptable maximum bias estimates. The filtering with higher cutoff values underestimates the bias (Fig. 2b, dark blue line on the bottom).

The whole maximum bias estimating procedure based on Eq. 4 may be omitted if the height of free-energy barrier is already known, for example, if it is experimentally estimated (real-life experiments).

Palassini and Ritort derived a bias estimator [33] that uses parameters of the external work distribution, instead the behavior of the free energy estimate itself. In our case, the application of their bias estimator may be difficult to implement because the external work distribution changes width along the reaction path.

## Maximum bias reduction

The fast polymer stretching generates a dissipation which inevitably introduces a bias into the Jarzynski PMF estimate. That bias can be reduced through the increase in the number of pulling trajectories, or through the decrease of the pulling velocity [13], [16]. Unfortunately, both approaches are computationally unfeasible; a number of samples required to decrease the estimate’s bias to an acceptable level, for example, less than 1 *k_B_T*, may be larger than the number of trajectories experimentalist can expect to generate using SMD or AFM [10], [16], and the decrease of the pulling velocity is computationally always expensive. Therefore, a different approach has to be applied to achieve a desired quality of reconstruction. One approach may be the increase of the probability of generating work samples with a reduced dissipation, while maintaining the same pulling velocity. A number of methods was devised to increase that probability, i.e., to improve the external work sampling. We gave an overview of these methods in our previous publications (see Discussions in Refs. [13], [34]). In this work we followed the same idea, namely, we wanted to intentionally broaden the external work distribution in a controlled manner in attempt to increase the probability of generating work samples with a small dissipation. This approach inevitably increases the error of the mean work, but also increases the number of low dissipation samples emphasized by the Jarzynski equality [13].

The work distribution can be unintentionally broadened by the imperfections of the experimental setup, but its width can be purposely increased through the introduction of the random fluctuations of the pulling spring. Those fluctuations can be applied via symmetrically distributed random perturbations of the pulling point (cantilever), i.e., through the intentionally added zero mean external noise. The pulling spring then directly transfers that noise to the pulled point (the pulled terminus of a polymer). The pulling point in that case follows the stochastic pulling protocol
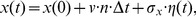
(5)in contrast to the normal pulling where the pulling point moves deterministically



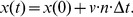
(6)In this scheme, the stochastic component is guided by the distribution function *η*(*t*) with a standard deviation *σ_x_*. We choose to apply the normal distribution to guide the stochastic perturbation because it is the most prevalent distribution in nature and its analytical treatment is straightforward. Therefore, the external noise thus applied increases the standard deviation of the pulled point through the fluctuations of the pulling spring. The increased deviation of the protein’s position increases the fluctuation of the external work and consequently improves the external work sampling. This approach has to omit the external noise at the very sampling moments to allow the exact external work calculation through the weighted histogram protocol (Eq. 2). If the noise component is present during the sampling moments, the external work exhibits additional fluctuations, and make the external work calculation less precise, and the whole PMF reconstruction procedure impractical. Without the omission, the calculated work can be an approximated if it is assumed that the pulling point moves deterministically. However, that approach does not accurately calculate the external work. In our implementation, the external work calculation is exact because at the sampling moments, the pulling point has the same value *x*(*t*) it should have if it would be moving completely deterministically (Eq. 6).

The work generated by the stochastic movement of the pulling point can be treated as a function of the random spring extension *X* and the spring stiffness *k* as 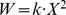. For a given spring extension distribution function *f*(*x*), the distribution function of the random work *W* is [35]

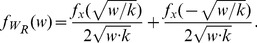
(7)


If the external noise is applied with the every time step of the simulation, its standard deviation can be given as a multiple of the standard deviation of the position of the pulled particle, caused by the thermal fluctuations of the polymer and its environment. We want to express the standard deviation of the pulling cantilever *σ_x_*, as a multiple of the standard deviation *σ_r_* of the pulled atom caused by thermal fluctuations. For a known diffusion coefficient *D*, the standard deviation of the pulled particle is, according to the theory of Brownian motion, equal to 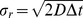, [17], [22], [36]. Following that, we express the standard deviation of the noise applied to the pulling point as 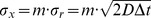, with *m* being the *noise multiplication factor*, i.e., *noise amplitude*. To calculate the required noise amplitude *m*, we used 3.5 · 10^−9^ m^2^s^−1^ for the diffusion coefficient *D*. That value is based on the diffusion coefficient estimate of Park et al. [16]. They estimated maximum *D* to be 2.7 · 10^−9^ m^2^s^−1^, using their SMD data based on the normal pulling protocol. We increased that value 30% to account for the maximum width of the external work. That increase does not change the effective noise amplitude because a lower value of *D* would only increase the required noise amplitude *m*.

When the random variation of the pulling point 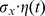 is normally distributed, the distribution of the external random work (Eq. 7) belongs to the family of *chi-square* functions
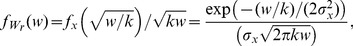
(8)with a standard deviation



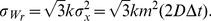
(9)The work calculation via the weighted histogram protocol requires the noise omission at the very sampling moments, as we explained previously [13]. The value of the pulling coordinate *x*(*t*) at those moments is the same as in the case of the normal (non stochastic) pulling. However, the effective work amplitude (standard deviation of the stochastic external work) averaged over *n* time steps between two sampling points is approximately 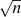 times smaller than the standard deviation of the instantaneous work fluctuation. Therefore, the effective noise is expressed as the root mean square deviation of the instantaneous noise over *n* time steps; in our case with the 10 m/s pulling, *n* is 200. Consequently, the effective deviation of the external random work is
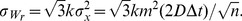
(10)


Here we assume that the effective work distribution, follows the chi-square distribution. This assumption is based on the fact that the additional external work has a much larger amplitude, i.e. standard deviation, than the internal, natural noise. We used a stiff spring to pull the polymer (stiff spring reduces the relaxation time of a polymer-spring system, see Ref. [16]), but we also assumed that the external work partly deviates from the Gaussian behavior for large noise amplitudes. Our simulation experiments confirmed this assumption; see for example Fig. A1c in the Appendix S1.

In our previous paper [13], based on a simple one-dimensional Brownian motion protocol, we showed that in order to reduce the Jarzynski bias, with a limited number of samples, the standard deviation of the external random work 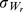 should be close to the bias of the mean work 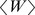 based on the normal, non-stochastic pulling protocol. That approach offers a much faster estimate convergence than the normal, constant velocity pulling. With an infinite number of samples, a Jarzynski equality estimate inevitably converges, even if the standard deviation is larger than the bias due to asymmetry of the external work distribution. This is the key property of the Jarzynski equality.

Previously, we only dealt with the improvement of the convergence of the Jarzynski PMF estimator [13], while in the present study we want to show that the increased work variance can improve the PMF calculation based on the second cumulant expansion formula also. A normal distribution of the external work is required for the application of this formula (moments higher than the second have to be zero). That means that the pulling point (pulling spring) distribution must induce a normal distribution to the external work. Although the stiff spring [16] imposes a normal distribution on the external work, the high amplitude external noise may produce a distribution which deviates from the Gaussian shape. To address that issue we decided to apply a pulling point distribution which inevitably imposes a normal distribution to the external work.

If *W* is the external work and *X* is the random spring extension with a distribution function *f_X_*(*x*), then *W* can be written as *W*  =  *g*(*X*). The probability density of the external work *f_W_*(*w*) is obtained as the first derivative of the probability function *F_W_*(*w*). That derivative can be expressed via the probability density function of the spring extension *f_X_*(*x*) and the function *h*, which is the inverse of the function *g*(*X*), *h*  =  *g* ^−1^,
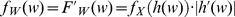
In our case, the function *g*(*X*) is a quadratic function (*W*  =  *k·X* ^2^). Therefore, we can write



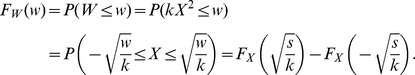
The probability density function is,



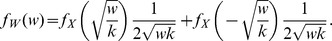
The function *f_W_*(*w*) has to be a normal distribution, which means that *f_X_*(*x*) has to be
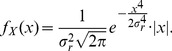
(11)Consequently *f_W_*(*w*) is



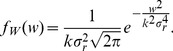
(12)To generate the desired density *f_X_*(*x*) we applied a simple elimination method (Rejection algorithm). The details of this scheme are given in the Appendix S1 (Elimination scheme for generating random numbers - rejection algorithm).

When the second-order cumulant expansion approximation is used, the amplitude of the external noise has to be smaller than the amplitude of the noise applied with the Jarzynski equality. The amplitude is smaller because the second order cumulant has a quadratic dependence on the work deviation, (the *second cumulant* is 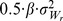), while 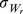 itself has a quadratic dependence on the noise amplitude. Therefore, the second cumulant expansion has a fourth-degree dependence on the noise amplitude *m* (see 1, Fig. A2 in Appendix S1). The second reason why the cumulant expansion converges faster is in the behavior of the external work. If the external work is normally distributed, all cumulants after the second are identical to zero. Those cumulants, if present, would give a positive contribution to the cumulant expansion sum and thus reduce the convergence rate.

### Constant velocity pulling with the additional constant variance noise

To test the ability of the external noise to improve the PMF calculation we followed the methodology described in the Section IV of our previous paper [13]. We tested a simple stochastic protocol that adds the constant variance noise during the constant velocity pulling. We applied that protocol to stretch deca-alanin. The noise multiplication factor *m* was calculated via Eq. 10. If the standard deviation of the external work 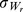 is equivalent to the mean work *bias* (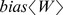), based on the normal pulling, the noise amplitude *m* should be equal to (see Eq. 10 in [13])
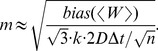
(13)


The crucial element of this calculation is the knowledge of the maximum mean work 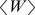 bias. We already depicted the procedure for the Jarzynski bias estimation (see Chapter 3 and Fig. 2). The bias of the mean work can be easily calculated as the sum of the Jarzynski bias and the difference between the mean work and the Jarzynski PMF estimate (the mean work and the initial Jarzynski estimate are known and both are based on the same set of pulling trajectories). To account for the variable noise amplitude (that issue will be explained later, in the section dealing with the noise modulation), we multiplied the expression for the denominator 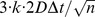 with (0.74)^2^. 0.74 is the average value of the noise guiding function. We squared it because the amplitude-modulated noise has a quadratic dependence on the noise amplitude.

We tested this stochastic perturbation protocol with the 10 m/s pulling using six different noise amplitudes: 160, 170, 180, 190, 200 and 210. The noise amplitude *m* equal to 160 corresponds to the maximum mean work bias of 28 *k_B_T; m* equal to 170 corresponds to the maximum bias of 32 *k_B_T*; *m* equal to 180 corresponds to maximum bias of 36 k_B_T, *m* equal to 190 corresponds to the maximum bias of 40 *k_B_T*, *m* equal to 200 corresponds to the maximum bias of 45 *k_B_T*, and *m* equal to 210 corresponds to the maximum bias of 50 *k_B_T* (see Appendix S1, Chapter - Noise amplitudes, Fig. A2 in Appendix S1). The amplitudes values which exactly correspond to the above given biases slightly differ from the given amplitudes; we rounded the amplitudes to a closest decade for easier analysis. We used these amplitudes to test the behavior of the perturbation protocol when the amplitude of the external noise precisely corresponds to the maximum bias, and to test the protocol when the noise has higher and lower amplitudes due to the imperfection of the bias estimation protocol. We also used different noise amplitudes because we wanted to examine how the PMF estimator behaves when the number of trajectories per reconstruction gets increased. With each noise amplitude, we performed 10,000 simulations and calculated corresponding PMF estimates using the Jarzynski equality (Eq. 2).

**Figure 3:**
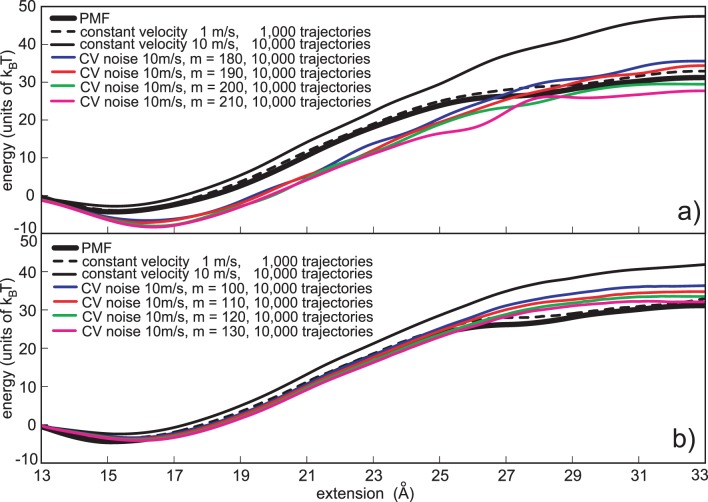
Constant variance noise based reconstructions. a) Jarzynski PMF estimates based on the constant variance noise protocol. The Gaussian noise was applied with the 10 m/s pulling velocity. Four noise amplitudes are depicted, *m* = 180, 190, 200 and 210. b) Cumulant expansion PMF estimates based on the constant variance noise protocol. The Chi-square noise was applied with the 10 m/s pulling velocity. Four noise amplitudes are depicted, *m* = 100, 110, 120 and 130. The estimates based on the normal pulling (1 m/s and 10 m/s) are given for the comparison. In each case depicted the computational cost is the same, i.e., it is analogous to the cost required to generate 10,000 trajectories using normal pulling and 10 m/s velocity.

Fig. 3a depicts PMF estimates based on the constant variance stochastic perturbation protocol, reconstructed with the Jarzynski equality. Besides four reconstructions based on the stochastic pulling protocols (based on four highest Gussian noise amplitudes, *m* = 180 to 210), two reconstructions based on the normal pulling protocol (1 m/s and 10 m/s), are also shown. It is obvious that the external noise helps in decreasing the maximum 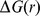 overestimate (the maximum Jarzynski bias, at the end of the reaction path, is reduced to zero, using the same number of trajectories as with the normal pulling), but it also introduces a significant underestimate along the reaction path, regardless the noise amplitude *m*. That means that the external noise with the constant variance is not able to consistently improve the Jarzynski based PMF estimation. The similar underestimate was observed in the real world, when the optical tweezers were used to probe a single RNA hairpin [37]. We will addressed the underestimate in the last Chapter of the Appendix S1.

We also applied the stochastic perturbation protocol with the second cumulant expansion formula (Eq. 3). To calculate the required noise amplitude we followed a simple assumption; if the mean work bias (the difference between the mean work and PMF) is equal to the second cumulant of the external work, 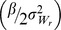, the bias of the estimate based on the second-order cumulant expansion formula will be annulled. In this case also the effective work variation has to be expressed as the root mean square deviation because the external noise has to be omitted at the very sampling moments. Therefore, the work deviation has to be divided by 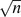, as with the Jarzynski noise, where *n* is the number of steps between any two sampling points, 200 in the case of 10 m/s pulling.
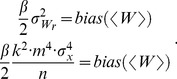
(14)If the second cumulant 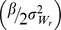 is equal to the mean work bias, the noise amplitude *m* is



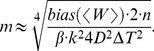
(15)With the cumulant expansion formula and with the 10 m/s pulling, the mean work bias is 37 *k_B_T*, therefore, the noise amplitude *m* has to be 100 (Eq. 15). We used higher and lower noise amplitudes, *m* = 80 to 130, in steps of 10, to test how they affect the reconstruction (*m* = 80 corresponds to the maximum bias of 15 *k_B_T*, and *m* = 130 corresponds to the maximum bias of 110 *k_B_T*, see Fig. A2 in the Appendix S1). We used noise amplitudes which correspond to very high bias values, much higher than the real bias, because the polymer itself dampness the random spring fluctuations. Besides that, the work distribution is never an ideal Gaussian. It has “fat” tails, which move the estimate up. Furthermore, the cumulant expansion formula is sensitive only to the variance of the external work, which moves more slowly than the width of the work distribution. Thus, the noise amplitude has to be higher than estimated by Eq. 15. The noise modulation protocols also require higher noise amplitudes because they reduce the efficiency of the external noise. We will address them later.

Fig. 3b shows PMF estimates based on the stochastic perturbation protocol, calculated with the second-order cumulant-expansion formula (Eq. 3). Besides four reconstructions based on the stochastic pulling protocols (based on four highest chi-square noise amplitudes, *m* = 100 to 130) two reconstructions based on the normal pulling protocol (1 m/s and 10 m/s), are also shown. When compared to the Jarzynski estimates, it is clear that these estimates have lower biases, and noticeably smaller underestimates. Their noise amplitudes, although smaller than the amplitudes applied with the Gaussian noise and the Jarzynski averaging, are more efficient in improving the PMF calculation. The highest noise amplitude (*m* = 130, magenta line) is the most successful in reducing the bias with only a small, but noticeable underestimate.

It is obvious that the external noise with a constant variance efficiently reduces the bias when the cumulant-expansion formula is used, but it is not an appropriate tool to improve the convergence of the Jarzynski PMF estimator. The high noise at the beginning of the reaction path may force a polymer to suddenly unfold. That irregular unfolding is reflected as the PMF underestimate because the Jarzynski equality emphasizes low dissipation work trajectories. The external noise, besides spreading the external work distribution, also slightly reduces the mean value of the external work (see Fig. A1 in the Appendix S1). That happens because the polymer perturbed with the additional external noise easier crosses the free energy barrier. The second-order cumulant-expansion formula converges faster, with smaller noise amplitudes than the Jarzynski equality, but it cannot avoid a small PMF underestimation, especially with higher noise amplitudes. All that implies that the external noise has to be adapted to the bias behavior along the reaction path. We previously showed that the information on the bias evolution along the reaction path is contained in the fluctuations of a Jarzynski estimate [13]. That behavior can be used to adapt the external noise to the bias.

### Bias behavior along the reaction path

A Jarzynski based PMF estimate, when dissipation is high and the number of work samples is limited, contains a bias [10], [16]. The bias appears because the usually short pulling time does not allow the complete release of the thermal energy accumulated in the system. The narrow work distribution is not able to efficiently sample the external work, which means that on average, the Jarzynski PMF bias is an increasing function of the pulling coordinate. The increase is not uniform because the underlying PMF is not a simple, linear function. Gore, et al. [10], as well as Zuckerman and Woolf [31], [32], showed that there is a direct, nonlinear relation between the variance of the estimate and its bias (see Eq. 4). Therefore, it can be assumed that the behavior of the estimate’s fluctuation roughly depicts the bias evolution along the reaction path [10], [13].

**Figure 4:**
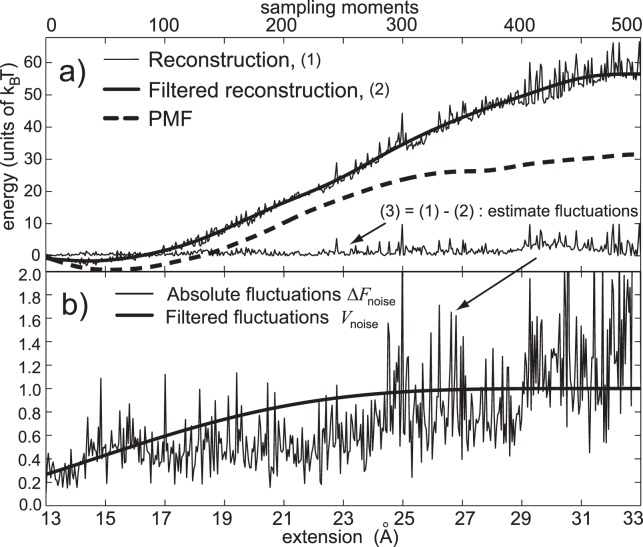
Jarzynski PMF estimate fluctuations analysis. a) Extraction of the estimate fluctuations by subtracting the filtered estimate from the original Jarzynski estimate. b) Creation of the function *V_noise_*(*r*) by filtering out the estimate fluctuations (as an average extracted from 5 reconstructions based on 10 different trajectories each).

The fluctuations of a Jarzynski based PMF estimate can be extracted by subtracting the smoothed version of the estimate from the original, Jarzynski one (see the bottom line in Fig. 1a and Fig. 4a). The first step in the fluctuation extraction is the calculation of the smoothed estimate. To get the smoothed estimate, we again applied the 5^th^ order Butterworth filter. The limited sampling produces a higher variance of the Jarzynski estimate, especially at the end of the reaction path where the accumulated energy is highest. To properly access the fluctuations we used a lower cutoff frequency (0.02 of the sampling frequency, for 500 reconstruction points per estimate), and to get a statistical overview, we calculated 5 reconstructions; each based on 10 separate fast puling trajectories. We did not use more than 5 PMF reconstructions because we wanted to reduce the computational costs. We used a slightly higher cutoff frequency because we wanted to extract high amplitude fluctuations caused by the limited sampling. The computational cost to produce those 50 work trajectories is negligible in comparison to the total computational cost (10,000 fast pulling trajectories). Besides, it was shown that the bias in this kind of experiments falls rather slowly with the increase in the number of samples [10], [16].

The normalized average of 5 sets of estimate fluctuations, 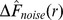 (Fig. 4b), obtained following the above procedure, can be seen as a rough description of the bias behavior along the reaction path. However, that description is itself very noisy. That noise could hardly be an artifact of the underlying potential because sudden changes of PMF along the reaction path would generate forces much higher than the forces expected from a biological system. As we already explained before (Chapter 3), those high frequency fluctuations were generated by the inefficient sampling and high dissipation, which means that they have to be filtered out. As before, we applied the low-pass Butterworth digital filter to perform that task. The cutoff frequency was lowered to 0.004 due to the rough nature of the extracted bias fluctuations (we came to that cutoff value by observing the shape of the filtered output). The absolute, normalized, average version of those fluctuations 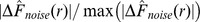 and their smoothed, normalized variant *V_noise_*(*r*) are both shown in Fig. 4b. We moved the guiding function *V_noise_(r)* down to 0 and normalized it to avoid the high external noise at the beginning of the reaction path.

The *V_noise_*(*r*) function, when compared to the shapes of the original and reconstructed potentials shows that our assumption is correct, i.e. that the amplitude of the estimate fluctuations roughly follows the evolution of the bias [13]. The exact relationship would be hard to obtain because dissipation and the bias, both depend on the underlying potential which shape is unknown before the experiment. The precise knowledge of the bias behavior along the reaction path would be sufficient to remove the bias from the PMF estimate, but that information is impossible to obtain with perturbations far from equilibrium and a small number of work trajectories.

## Two improvements of the stochastic perturbation protocol

In our previous paper [13] we introduced two adaptive stochastic perturbation (ASP) protocols which utilize the position dependent function *V_noise_*(*r*) to adjust the externally applied noise to the bias behavior. The first and more obvious protocol modulates the amplitude, i.e., the standard deviation of the external noise, so we named it the *amplitude modulation* (AM). The second protocol modifies the probability of the noise appearance during the stretching, which means that it deals with the frequency of the noise appearance, so we named it the *frequency modulation* (FM). This chapter gives a detailed description of their full-atom SMD implementations.

### Amplitude modulation of the external noise

The amplitude modulation protocol (AM), as it name implies, alters (modulates) the standard deviation *σ_x_* of the externally applied noise (stochastic fluctuation of the pulling point) during the polymer stretching. The standard deviation in that case is not constant during the perturbation; it depends on the pulling coordinate through the normalized function *V_noise_*(*t*):
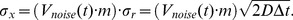
(16)This method controls the work performed by the external noise through the modulation of its amplitude via the position dependent function *V_noise_*. The current sampling time step *t* is used to extract the corresponding value of the function *V_noise_*. This approach is applied because the pulled point *r*, i.e. the protein itself, exhibits random fluctuations due to presence of both thermal and external noises. The random fluctuations prohibit the continuous change of the noise amplitude. Our aim was to change the function *V_noise_* continuously and deterministically throughout the perturbation, thus we opted to extract the values of the function *V_noise_* via the sampling time step *t*, instead via the pulled coordinate *r*. That means that the values of the function *V_noise_* are extracted using the position of the cantilever *x* (without noise being added), which is, itself, linearly dependent on the pulling moment *t*. This approach is acceptable because the stiff spring keeps both (pulled and the pulling) points in proximity. The function *V_noise_* changes value at the sampling moments and on those moments, the pulling point has deterministic values (the noise is not added when the external work is sampled, as we already explained). Therefore, the above equation should be read as
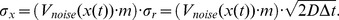
(17)When the polymer is stretched using the normal, constant velocity pulling the cantilever covers an equal distance between every two sampling steps. That distance is made of a large number of equally distributed (sub)steps. The stochastic perturbation assumes that the external noise is applied with each and every of those intermediate steps (except at the very sampling moments when the external noise is omitted). With the amplitude-modulated external noise, *σ_x_* is multiplied by the position dependent function *V_noise_*(*t*). Fig. A3a in the Appendix S1 depicts how the noise amplitude changes throughout a single pulling experiment. When the number of simulation steps between two sampling points is *n*, the average work performed by the amplitude-modulated external noise is
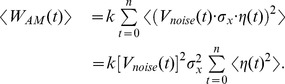
(18)The second line stems from the fact that the function *V_noise_* is constant between every two consecutive sampling moments. It is constant because the function (signal) *V_noise_* has the same resolution as the sampled external work, and the reconstruction (500 points along the reaction path). This derivation shows that the average random work of the amplitude modulate noise has a quadratic dependence on the function *V_noise_*.

We applied the AM protocol only to the fast pulling regime, i.e. to the 10 m/s velocity. To reconstruct PMF we applied both averaging schemes (Eqs. 2 and 3). The noise amplitudes for both sets of simulations (Gaussian and chi-square noise) were the same as with the constant noise perturbations experiments. To test the efficiency of the AM noise protocol we conducted 10,000 simulations with each noise amplitude.

**Figure 5:**
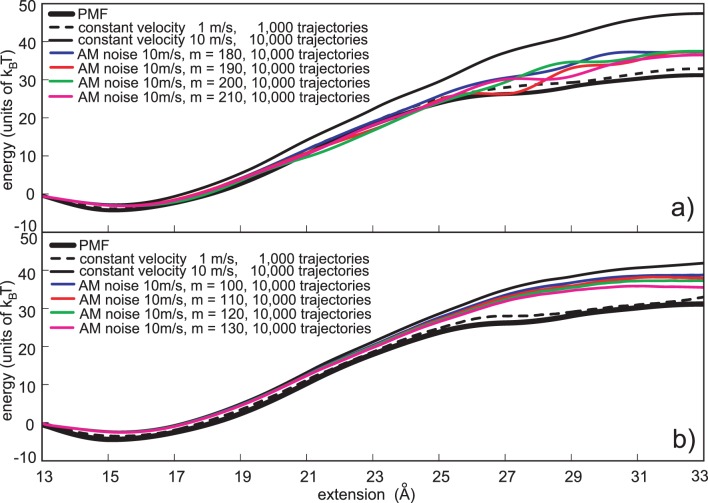
Amplitude-modulated (AM) noise based reconstructions. a) Jarzynski PMF estimates based on the AM protocol. The Gaussian noise was applied with the 10 m/s pulling velocity, using four noise amplitudes, *m* = 180, 190, 200 and 210. b) Second order cumulant expansion PMF estimates based on the AM protocol. The chi-square noise was applied with the 10 m/s pulling velocity using 4 different noise amplitudes, *m* = 100, 110, 120 and 130. The estimates based on the normal pulling (1 m/s and 10 m/s) are given for the comparison. In each case depicted the computational cost is the same, i.e., it is analogous to the cost required to generate 10,000 trajectories using the normal pulling and 10 m/s velocity.

Fig. 5a shows that the AM noise protocol coupled to the Jarzynski equality effectively reduces both the maximum PMF bias and the PMF underestimate. The AM protocol improves the PMF reconstruction even when the maximum bias is underestimated, i.e. when the required noise amplitude is underestimated. Of all six noise amplitudes (four depicted in Fig. 5) the highest noise amplitude (*m* = 210) was the most efficient. It was able to efficiently reduce the maximum bias, without a pronounced underestimate. The statistical analysis, given later in the paper, shows that the quality of the reconstruction obtained with only 100 trajectories and the AM noise protocol cannot be achieved with the normal pulling and 10,000 trajectories.

The comparison of Figures 3a and 5a shows that the AM noise is less effective in the maximum bias reduction than the constant variance noise. That is to be expected, because the reduced amplitude of AM noise at the beginning of the simulation reduces the probability of generating trajectories with a minimal work and thus reduces the overall probability of generating work samples with a minimal dissipation. That also implies that the amplitude of the external noise has to be increased, when the noise modulation is used, in order to achieve the bias reduction analogous to the reduction produced with the constant variance noise.

The AM perturbation protocol was also tested with the cumulant expansion formula. We used the same noise distribution (chi-square) and the same noise amplitudes (80 to 130) as with the constant noise perturbation, see Chapter 4.1. We also applied the AM protocol with the same *V_noise_* function as with the trajectories intended to be reconstructed with the Jarzynski equality. This approach can be accepted because the function *V_noise_* is only a rough descriptor of the bias. Fig. 5b shows the results of this approach. The AM protocol reduces the bias, although not as effectively as the constant variance noise, but it also successfully eliminates the underestimate.

Figure 5 (panels a and b) shows that the AM protocol can effectively reduce the bias of a PMF estimate caused by a far from equilibrium perturbation. The final outputs are of a similar quality for both averaging schemes, although the applied noise amplitudes are not equal. That happens because the cumulant-expansion formula has initially smaller bias and converges faster than the Jarzynski equality. However, that also means that the AM protocol more effectively reduces the bias of the Jarzynski estimator, than the bias of the cumulant expansion formula.

### Frequency modulation of the external noise

Stochastic fluctuations of an unperturbed system are caused by the system's own internal energy, and by the energy of the surrounding thermal bath. Their joint variance is primarily influenced by the properties of the heath bath because the observed system is usually much smaller than the bath. If the fluctuations have a clearly defined distribution, then the energy they transfer to the system is determined by either their standard deviation, or the duration of a time step between their successive applications [30], [38]. The change of the time step is, in essence, the change of the frequency of fluctuations. Regardless of the fluctuations’ nature (internal, or external) the control of their frequency determines the amount of energy they transfer to the system. In the previous chapter, we dealt with the amplitude modulation of the external noise and in this we are going to address the modulation of its frequency (FM noise).

The energy pumped into a system by an energy-carrying signal depends, besides its amplitude and shape, on its frequency also. That frequency can be defined as the number of energy *packets* received by the system between consecutive sampling moments [30], [38]. The more energy packets are generated per unit of time, the more energy is transferred to the system. It is important to remember that the energy carrying signal does not have to be deterministic, its nature can be stochastic (such a signal can be seen as a sum of infinitely many harmonic components with a flat power density spectrum [29]). The instantaneous value of that signal can be hard to obtain, but its average value is often easily calculated or measured. In our case, if the number of simulation time steps between two sampling points is *n*, the average energy pumped into the system, i.e., the average work performed by the external stochastic signal [38] is equal to:
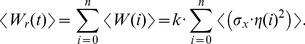
(19)This relation shows that the average work 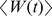 directly depends on the number of noise samples *n*. Consequently, the amount of the stochastic work can be controlled by the alteration of that number. The normalized function *V_noise_*, which we already used to control the amplitude of the external noise, is ideally suited for that task. When this function is equal to 1, the noise should be applied without any constraints. When this function is equal to 0, the external noise should not be applied at all, i.e., the stochastic fluctuations should come from the system's internal energy and the thermal environment only. When the current value of this function is between those two extremes (between 0 and 1), the number of noise applications during *n* simulation time steps should be as close to 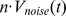 as possible, where *t* corresponds to the current sampling time step. The question remains, what is the most efficient way to distribute 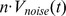 random impulses over the *n* time steps? That issue becomes emphasized when the external noise has to be applied with a real-life experimental setup, for example with AFM. To address that issue, we devised a method which randomly determines whether the external noise with the constant variance is going to be applied, or not. The method uses an additional generator of uniformly distributed random numbers between 0 and 1, and compares its output *R*(*t*) to the current value of the function 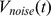. The constant variance noise is applied only if that output is smaller or equal to the current value of the function 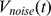. This method should, on average, generate 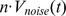 random perturbations over *n* simulation time steps between two sampling points. The very number of random impulses per particular trajectory may differ from 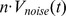, but it should be very close to that value if the number of simulation time steps between two sampling points is large enough to allow the random number generator to produce enough numbers recognized as uniformly distributed. Fig. A3b in the Appendix S1 depicts the behavior of the FM noise protocol during the stretching. The average work performed by the frequency-modulated external noise is therefore
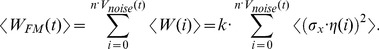
(20)The true comparison between the two modulation protocols, AM and FM, requires the comparison of their average works on equal terms. That can be done if the average work of the FM noise 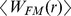, is expressed through the summation over *n* simulation time steps, instead of 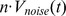 steps (for the average work of the AM noise see Eq. 18). To make things clear, we can imagine that the generator which produces the stochastic perturbation of the pulling point produces random fluctuations with every time step, regardless of the current values of the functions *V_noise_* and *R*(*t*). Its output is transferred to the pulling cantilever only if the random number *R*(*t*) is larger that the current value of the function *V_noise_*(*t*). Knowing that, we can express the random work of the FM noise through the random work of the uniformly applied noise with the constant variance (Eq. 19). Their ratio is, on average, 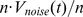. Therefore, the random work of the FM noise can be written as
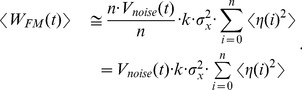
(21)When the number of steps *n*, between two sampling points, is large enough (as in our case), the average random work of the FM noise is linearly dependent on the function *V_noise_*, as opposed to the AM random work which has a quadratic dependence on *V_noise_* (Eq. 18). If the number of samples is large enough, the number of random impulses will be very close to *n*·*V_n_*(*r*). If the number of samples is smaller, the number of random impulse may significantly deviate from that value. Simply speaking, the average amount of energy the FM noise transfers to the system is directly proportional to the function *V_noise_*(*t*). That implies that the FM protocol more powerfully transfers noise to the polymer because it does not square the normalized function *V_noise_*(*t*).

We tested this method with the exponential averaging (Eq. 2) and with the second cumulant expansion formula (Eq. 3) using the same simulation parameters as with the amplitude modulation (see Chapter 5.1).

**Figure 6:**
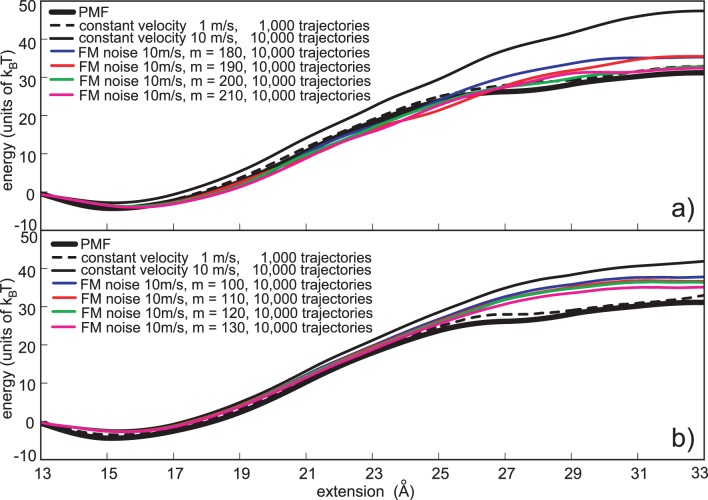
Frequency-modulated (FM) noise based reconstructions. a) Jarzynski PMF estimates based on the FM stochastic perturbation protocol. The external Gaussian noise was applied with the 10 m/s pulling velocity, using four noise amplitudes, m = 180, 190, 200 and 210. b) Second order cumulant expansion PMF estimates based on the FM stochastic perturbation protocol. The chi-square noise was applied to 10 m/s pulling velocity using 4 different noise amplitudes, *m* = 100, 110, 120 and 130. The estimates based on the normal pulling (1 m/s and 10 m/s) are given for the comparison. In each case depicted the computational cost is the same, i.e., it is analogous to the cost required to generate 10,000 trajectories using the normal pulling and 10 m/s velocity.

Fig. 6a depicts the efficiency of the FM noise with the Jarzynski averaging scheme. As with the constant and the AM noise, we used six noise amplitudes (*m* was between 160 and 210), but depicted only results based on highest four (all six were used in the statistical analysis). The amplitude next to highest, *m* = 200, was the most successful in improving the reconstruction. It was able to efficiently reduce the bias, and keep the underestimate under control. The three other noise amplitudes depicted (180, 190 and 210) were also able to improve the PMF calculation, although not as effectively as *m* = 200. All four reconstruction show small underestimates. Those underestimates can be dealt with if all their pulling trajectories are closely examined. The ones that strongly deviate from the general behavior should be generated again. The new Jarzynski calculations, with those trajectories replaced, should produce slightly better PMF estimates.

The differences in *V_noise_* interpretation between the two modulation protocols become apparent when Figures 5a and 6a are compared. The AM protocol “squares” the guiding function *V_noise_*, while the FM noise uses the original *V_noise_*. Therefore, the FM noise more efficiently reduces the maximum bias, but also introduces a small underestimate.

The influence of the FM noise on the cumulant expansion based PMF estimation is depicted in Fig. 6b. The simulation parameters were the same as with the amplitude modulation protocol (*m* was between 100 and 130) and the pulling spring again followed the chi-square distribution as in the two previous cases (constant amplitude noise and AM noise). The highest noise amplitude, *m* = 130, was the most successful in improving the PMF reconstruction. It reduced the overestimate to less than half its normal pulling value, without introducing an underestimate. The three smaller noise amplitudes were also able to reduces the bias, although less effectively than *m* = 130. As with the Jarzynski equality, the comparison of Figs. 5b and 6b shows that the FM noise applied with the cumulate expansion formula is more powerful in reducing the PMF overestimate, than the AM noise.

## Comparison of the efficiency of the three stochastic perturbation protocols

The PMF estimates based on trajectories generated with the additional external noise converge faster than the estimates based on the normal pulling only. The question remains, which of the three stochastic protocols is the most successful in improving the PMF calculation? The relative RMSD (root mean square deviation, expressed as the percent of the potential barrier height) and the computational cost per reconstruction are good measures of the efficiency of the applied perturbation protocols. For *N* reconstruction points along PMF, the relative RMSD is
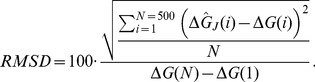
(22)


**Figure 7:**
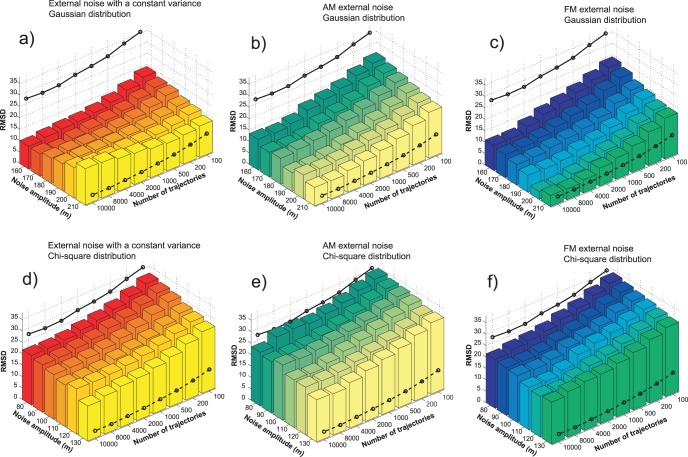
PMF reconstruction quality expressed as the relative RMSD, for the reconstructions calculated with the Jarzynski equality. The pulling trajectories were generated using the three stochastic perturbation protocols (constant variance noise, AM noise and FM noise). The noise amplitude *m* was in the range between 160 and 210, for the Gaussian distribution of the pulling point (panels *a*, *b* and *c*), and between 80 and 130, for the chi-square distribution of the pulling point (panels *d*, *e* and *f*). The full line represents the reconstruction quality for the normal pulling and 10 m/s velocity. The dashed line is the reconstruction quality for the normal pulling and 1 m/s velocity.

**Figure 8:**
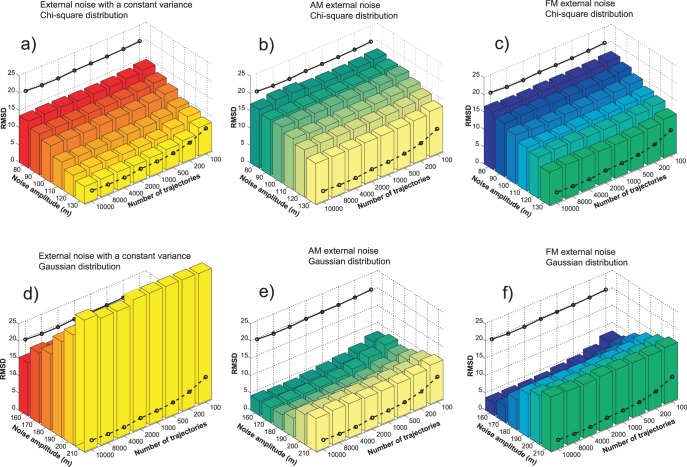
PMF reconstruction quality expressed as the relative RMSD, for the reconstructions calculated with the cumulant expansion formula. The pulling trajectories were generated using the three stochastic perturbation protocols (constant variance noise, AM noise and FM noise). The noise amplitude *m* was in the range between 80 and 130, for the chi-square distribution of the pulling point (panels *a*, *b* and *c*), and between 160 and 210, for the Gaussian distribution of the pulling point (panels *d*, *e* and *f*). The full line represents the reconstruction quality for the normal pulling and 10 m/s velocity. The dashed line is the reconstruction quality for the normal pulling and 1 m/s velocity.

With each simulation setup we conducted 10,000 trajectories (except with the 1 m/s pulling with which we performed 1000 simulations only). The PMF reconstructions were carried out using all 10,000 trajectories and all their smaller non-overlapping subsets (100, 200, 500, 1000, 2000, 4000, 8000 trajectories). With the Gaussian distribution, we used the noise amplitude *m* in the range between 160 and 210, and with the chi-square distribution, we used *m* in the range between 80 and 130. The RMSD analysis was performed on each PMF reconstruction. The results of the RMSD analysis of the three stochastic protocols are depicted in Figs. 7 and 8. Fig. 7 depicts RMSDs for PMFs based on both set of simulations (Gaussian and chi-square) calculated using the Jarzynski equality, and Fig. 8 depicts analogous RMSDs of PMF estimates produced with the cumulant expansion formula. The computational cost is equivalent for each point along the number-of-trajectories axis. It is expressed as the number of trajectories generated using the 10 m/s velocity. That means that for the 1 m/s pulling, the number of trajectories per reconstruction is 10 times smaller than the values depicted in the figures. Critics may argue that with the external noise, we use the random number generator, which increases the computational cost, but that increase is negligible when compared to the total cost of the MD integration. We also used 50 trajectories to calculate the bias and the *V_noise_* function, but that cost is also very small in comparison to the overall computational cost to generate 10,000 trajectories.

Although we applied two different types of the external noise distribution (Gaussian and chi-square), we produced PMF estimates using both averaging schemes (Eqs. 2 and 3). The first set of simulations was intended for the Jarzynski equality averaging, but we applied the cumulant expansion formula on it also. We did the same with the simulations intended for cumulant expansion formula, i.e., we used the Jarzynski averaging on them also.

The most obvious conclusion that can be drawn from Figs. 7 and 8 is that the external noise improves the PMF calculation. However, the two averaging schemes and the three types of the external noise have different influence on the reconstruction. The Jarzynski averaging produced the best results with the FM noise guided by the Gaussian distribution (Figs. 6a and 7c). Those results are closely followed by the results based on the AM noise guided by the same distribution (Figs 5a and 7b). The RMSDs of the PMF estimates based on the samples generated with the Gaussian FM noise, with amplitude *m = *200, are almost comparable to RMSDs of the estimates based on the 10 times slower pulling without the external noise (1 m/s). Their average difference is less than 5%.

The Jarzynski equality produced a lesser quality output with the trajectories generated using the chi-square noise (see Fig. 7, panels *d*, *e* and *f*). It was able to reduce the overestimate, but not as efficiently as with the higher amplitude Gaussian noise.

The cumulant expansion formula produced the best results with the chi-square noise, when that noise was not modulated (Fig. 8, panel *a* and Fig. 3b, the bias reduction is prominent, and the underestimate is almost nonexistent). That outcome is not as surprising as it may seem. The samples perturbed with the chi-square distribution were generated with the smaller noise amplitude, than the Gaussian noise samples. The smaller noise amplitude reduces the probability of a sudden jump at the beginning of the reaction path. Such jumps are responsible for the underestimation. We also used the stiff spring to pull the polymer. The stiff spring forces a polymer to maintain the normal distribution of the external work, even when perturbed far from equilibrium [16]. We used the stiff spring because the second order cumulant expansion formula converges much faster with the Gaussian distribution than with other distributions. However, with the external noise, the external work may deviate from the normal distribution, thus we imposed a normal distribution to the external noise through the chi-square distribution of the pulling point. It must be noted, that the chi-square distribution may be difficult to generate with a real-world experimental setup.

With the Gaussian noise based trajectories, the cumulant expansion formula produced PMF reconstructions with a low RMSD only with the AM stochastic protocol (see Fig. 8e). However, low RMSDs of those reconstructions may be misleading because they do not resemble deca-alanin’s PMF, except with the lowest noise amplitude (see Fig. A4b. in the Appendix S1). With the constant variance Gaussian noise and the FM Gaussian noise the cumulant expansion formula does not produce a satisfactory output (see Figs. 8d, 8f and Figs. A4a and A4c in the Appendix S1).

## Conclusion

This paper describes the three perturbation protocols we have developed in an attempt to improve the potential of mean force reconstruction in real world and simulated single molecule manipulation experiments. The protocols improve the PMF calculation by improving the external work sampling through the widening of external work distribution. The work distribution gets widened through the application of the external noise, which itself gets introduced through the random fluctuations of the pulling spring. One way to interpret the external noise is to imagine that it comes from an additional heath bath directly coupled to a polymer being examined. That “heat bath” is completely independent from the solvent the perturbed system is immersed in. It transfers random fluctuations only along the pulling vector and its diffusion coefficient is variable and depends only on the reaction coordinate.

The application of the external noise requires the knowledge of the maximum bias. To estimate it, we followed the work of Gore et al. [10] and Zuckerman and Wolf [31], [32]. They produced a relation able to connect the fluctuations of a Jarzynski estimate to its bias under the assumption that the external work fluctuations follow the fluctuation-dissipation relationship [18]. That protocol may slightly overestimate the bias, but we showed that it is not an issue because the noise modulation protocols themselves decrease the noise efficiency and thus require noise amplitudes somewhat higher than the ones that ideally fit the true maximum bias.

We used two averaging schemes to calculate the PMF estimates, the Jarzynski equality and the second-order cumulant-expansion formula. Consequently, we applied two different external work distributions (Gaussian and chi-square) and a couple of different values of the noise amplitude *m*. Both reconstruction procedures, when the external noise has very high amplitude, produce an underestimate of PMF.

To address the PMF underestimate issue we introduced two noise modulation protocols that adapt the external noise to the bias behavior along the reaction path. Those two protocols are the amplitude modulation (AM) and the frequency modulation (FM). We also introduced the noise guiding function *V_noise_* to control the modulation protocols. That function was obtained through a (digital) filtration of the initial, constant velocity Jarzynski PMF estimates. With the amplitude modulation we used the normlized *V_noise_* function to multiply the amplitude of the external noise (standard deviation of the external noise), and with the frequency modulation we used this function to control the probability of the noise appearance.

The results of our experiments show that the modulated external noise is a proper tool to noticeably improve the PMF calculation with fast steered molecular dynamics experiments. The best overall results were achieved with the frequency-modulated noise and the Jarzynski equality. The quality of those reconstructions approach the quality of the reconstruction based on ten times slower pulling without the external noise. A similar quality can be achieved using the cumulant expansion formula and the constant variance noise. However, that noise has to follow a chi-square distribution, which may be difficult to generate in real-life experiments. Furthermore, we showed that the Jarzynski averaging is more flexible than the cumulant expansion averaging because the noise amplitude that produced the best PMF estimate with the Jarzynski averaging and the Gaussian noise was based on a bias estimate much closer to the real bias. The cumulant expansion averaging and the chi-square noise, on the other hand, require a noise amplitude based on a bias estimate much higher than the real bias to effectively reduce it.

The work depicted here is in a good correlation with the work of Kou and Xie [39]. They addressed the issue of an anomalous, subdifussion process with the fractional Gaussian noise guided by the memory kernel *K*(*t*). That approach is similar to our own research because a stretching simulation (SMD) with the additional noise can be perceived as an anomalous, subdifussion process in which the increased work variation increases the system’s correlation time. Therefore, our modulation protocols introduce the memory kernel *K*(*t*) via the function *V_noise_*. The model exhibits a dependence of fluctuations on the reaction coordinate even without the additional noise with the memory kernel *V_noise_* (see Park and Schulten, [16]).

One may wonder what would happen with an increase in the pulling velocity and with a consequential increase in dissipation. Will that increase require an analogous increase of the external noise amplitude? The external noise depends on two parameters, the maximum noise amplitude and the function *V_noise_*. The first parameter, the noise amplitude, is calculated via the maximum bias estimate. That calculation accounts for the noise omitted at the very sampling moments by reducing the effective work variance 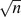 times, where *n* is the number of simulation time steps between two consecutive sampling moments (see Equations 10 to 15). The number of steps *n* gets reduced with the increase in the pulling velocity (if we assume that the simulation time step stays the same, regardless the pulling velocity). That means that the increase in the pulling velocity (and the consequential increase in the average bias) does not require a comparable increase of the noise amplitude (*n* used in Eqs. 13 and 15 will be reduced). For example, for the 20 m/s pulling, the number of steps between two sampling moments will be half of the steps used with the 10 m/s pulling. That means that the corresponding noise amplitudes calculated via Eqs. 13 and 15 have to be reduced by a factor of 2^1/4^ in comparison to the amplitudes used with the 2 times slower pulling (not counting the average work values).

Our work, although based on *in-silico* experiments, also addresses the influence of the real-world instrumental noise on the PMF calculation. The single molecule manipulation of biological molecules usually happens on physiological temperatures, and on those temperatures, not only the examined polymer, but the manipulation apparatus also, exhibits random fluctuations. The frequency response (dynamic regime) in real-world situations may be limited by the size of the manipulation apparatus (micrometer size of the AFM cantilever or optical tweezers beads). That size is, in some cases, three orders of magnitude larger than the length of the average biopolymer. That also means that thermal fluctuations of the manipulation apparatus can be much higher than the fluctuations of the perturbed molecule, and thus detrimental to the PMF calculation. Still, the influence of the thermal environment can be put to a good use. Namely, if the physical properties of the manipulation apparatus (stiffness of the cantilever, for example) can be controlled, the influence of the thermal environment could be adjusted, which means that the PMF calculation could be improved also [34]. Maragakis et al. [37] addressed the real-world instrumental noise present during the RNA hairpin stretching with optical tweezers. They observed that the fastest pulling experiments, the ones farthest-from-equilibrium, contain smallest amounts of noise. The lower pulling rates produce an underestimate of PMF if the instrumental noise is high. Those results are in agreement with our own observation that the excessive amounts of external noise may negatively affect the PMF calculation. In this work, we showed that the thermal fluctuations of the real-world manipulation tool, although supposedly small, could produce an underestimate when coupled to the Jarzynski equality or cumulant expansion formula. We also showed that those random fluctuations, if controlled, could significantly improve the PMF calculation. That is the most important result coming out of this study.

## Supporting Information

Appendix S1 (DOC)
