# Supplementary material for: On the Improvement of Free-Energy Calculation from Steered Molecular Dynamics Simulations Using Adaptive Stochastic Perturbation Protocols
Source: PLoS One. 2014 Sep 18;9(9):e101810. doi: 10.1371/journal.pone.0101810 (PMC4169427; doi:10.1371/journal.pone.0101810)
Supplement: Appendix S1 — (DOC) [file pone.0101810.s001.doc]

# On the improvement of free-energy calculation from steered molecular dynamics simulations using adaptive stochastic perturbation protocols

Ognjen Perišić^1,2,*^ and Hui Lu^1,2,*^

^1^Shanghai Institute of Medical Genetics, Shanghai Children’s Hospital,

Shanghai Jiaotong University,

1400 West Beijing Road,

Shanghai 200040, China

^2^Department of Bioengineering,

University of Illinois at Chicago,

820 S. Wood Street, W103,

Chicago, IL 60607-7052, U.S.A.

Tel.: (312) 413-2021, Fax: (312) 413-2018

^*^Corresponding authors:

ognjen.perisic@gmail.com, huilu@uic.edu

# Appendix

### Elimination scheme for generating random numbers - rejection algorithm

Let *Y* be a random number with a density function *g(y)* which can be generated, and let *f(y)* be the desired density function. The densities *f(y)* and *g(y)* have to be defined on the same set *D*, and they should satisfy the condition$f(y)\leq c\cdot g(y)$ for every *y* ∈ *D,* and for a given positive constant *c*. The elimination scheme then consists of two steps:

1. Generate a random number *Y* from *g(y)* and generate a uniformly distributed random number *U*.
2. If $U\leq f(Y)/cg(Y)$ accept *Y* as a random number having the density *f(y)*. Otherwise, repeat the step 1.

This algorithm generates random numbers with the desired density *f(y)*.

###

**Work distributions for the three protocols**

Figure A1

### Noise amplitudes

Figure A2

### Two modulation protocols

Figure A3

### PMF reconstructions generated with the cumulant expansion formula, based on the Gaussian distribution of the pulling point

Figure A4

### Theoretical analysis of the Jarzynski underestimate

When work fluctuations behave according to the fluctuation-dissipation theorem (FD) [1] the external work follows the Gaussian distribution. Knowing that, the probability of generating a trajectory with negative dissipation can be expressed as [2]

. (A.1)

When the external work does not behave according to the FD theorem, the work fluctuations can be represented as the sum of two independent random signals. The first signal can be imagined to follow the FD theorem, and thus have a normal distribution. The second signal can also be imagined to have a normal distribution, but with a variance which does not have a direct relationship to dissipation. We choose the normal distribution to guide the second signal (the external noise for example) because the sum of a large number of random variables/signals is approximately *Gaussian* (normal distribution) [3]. In that case, the variance of the additional fluctuation, i.e. noise, can be written as $\sigma_{\alpha}^{2}=\alpha^{2}\sigma_{X}^{2}$ with $\sigma_{X}^{2}=2\cdot\bar{W}_{dis}/\beta$ (the fluctuation-dissipation work variation). The convolution of two Gaussian probability density functions is a Gaussian distribution function

. (A.2)

This equation says that the sum of two normally distributed random variables (noises) has a normal distribution with a variance equivalent to the of variance of the equilibrium work fluctuations [1, 4] multiplied by the scaling factor $\left(1+\alpha^{2}\right)$. With the help of Eq. A.2 the probability of generating a trajectory with negative dissipative work can be expressed as

. (A.3)

A simple interpretation of this equation says that the work fluctuations which do not follow the FD theorem, increase the probability of generating work trajectories with a negative dissipation to the level of probability when the average dissipation is $\bar{W}_{dis}/\left(1+\alpha^{2}\right)$. Another interpretation of this result says that the influence of the additional noise is analogous to the increase of the heat bath temperature $\left(1+\alpha^{2}\right)$ times.

The influence of nonequilibrium fluctuations on the Jarzynski bias can be analyzed via stochastic variable $Y=\left\langle\exp\left(-\beta W\right)\right\rangle_{n}$. This approach is based on the approach used by Gore et al. and Zuckerman and Woolf, to analyze the behavior of Jarzynski estimator in the large *N* limit, when the work fluctuations follow FD [2, 5].

If the average dissipation is small and normally distributed, (a large dissipation would generate small exponential values [Eq. 10] which cannot be properly accessed) the stochastic variable *Y* has a mean

. (A.4)

and a variance

$\sigma_{Y}^{2}=\frac{Var\left(e^{-\beta W}\right)}{N}$. (A.5)

The Taylor expansion of the stochastic variable log(*Y*) around $\bar{Y}$produces:

(A.6)

When the higher terms of this expansion are discarded, the bias has a form which depends on three elements, the average dissipation $\bar{W}_{dis}$, the number of samples *N*, and the amount of the additional work noise *α*

. (A.7)

i.e.

. (A.8)

In the case of the Gaussian distribution of the external work (only in the near equilibrium regimes, where $\bar{W}_{dis}$ is small in comparison to $\beta^{-1}$), the bias is

. (A.9)

This equation has two elements; the first one, $-\alpha^{2}\bar{W}_{dis}$is dominant when dissipation is small compared to$\beta^{-1}$. The second element of the equation,, describes the overestimate. When the average dissipation $\bar{W}_{dis}$ is small, the bias is dominated by the first element of the equation. For *α* equivalent to zero, Eqs. A.8 and A.9 are identical to Eqs. 8 and 11 in Ref. [2].

Similarly, the variance of the Jarzynski estimator can be produced by expanding the stochastic variable log(*Y*) around $\bar{Y}$, i.e.,$\sigma^{2}\left(N\right)=\left\langle\left(\Delta G_{J}\left(N\right)-\left\langle\Delta G_{J}\left(N\right)\right\rangle\right)^{2}\right\rangle$

. (A.10)

When α is zero, the above equation is identical to Eq. 9 in [2]. The equation shows that the increased work variation directly reduces the variation of the Jarzynski estimator via the factor *exp*(2*βα^2^*$\bar{W}_{dis}$). Furthermore, the final expansion shows that the variance of the Jarzynski estimator corresponds to the variance of the variable exp(-*β*(1+ *α^2^*)$\bar{W}_{dis}$), which is smaller than the variance of *exp*(-*β*$\bar{W}_{dis}$).

This analysis shows that the Jarzynski equality coupled to a limited number of work samples can produce an underestimate when the perturbation is slow and work fluctuations do not follow the FD theorem. That underestimate can be roughly interpreted using the first element of Eqs A.7, A.8 and A.9, $-\alpha^{2}\bar{W}_{dis}$. With sufficiently many samples, the work distribution will move from an ideal Gaussian distribution to a distribution with asymmetric tails. Namely, it will have a fat tail on the right side, the side which corresponds to higher work values. That will happen because the left side tail corresponds to the spontaneous – random unfolding, and the right side to the forced unfolding. The distribution will be asymmetric because the spontaneous unfolding is usually less probable than the forced unfolding. It is less probable because the polymer has to spontaneously cross its own free energy barrier, which is usually higher than the amplitude of thermal fluctuations (*k_B_T*). The smaller probability of crossing the free energy barrier means that the tail on the left side (small or negative energies) will be shorter and less pronounced than the right side tail! Therefore, the asymmetric shape of the work distribution will produce an unbiased estimate of Δ*F* regardless the perturbation protocol, or the external work distribution width, only if enough samples are available. Zuckermann and Wolf addressed the influence of asymmetric tails on the free energy reconstruction in their 2004 publication [6]. Furthermore, with infinitely many samples, and the asymmetric work distribution, truncation of Eq. A6 would be impossible. That means that the first term that describes the underestimate would be compensated for with higher order terms.

**References**

[1] Callen HB, Welton T (1951) Irreversibility and generalized noise. Physical Review 83: 34-40.

[2] Gore J, Ritort F, Bustamante C (2003) Bias and error in estimates of equilibrium free-energy differences from nonequilibrium measurements. Proc Natl Acad Sci 100: 12564-12569.

[3] Athanasios P (1991) Probability, Random variables and Stochastic processes. McGraw-Hill, New York.

[4] Hermans J (1991) Simple analysis of noise and hysteresis in (slow-growth) free energy simulations. Journal of Physical Chemistry 95: 9029-9032.

[5] Zuckerman DM, Woolf TB (2002) Theory of a Systematic Computational Error in Free Energy Differences. Physical Review Letters 89: 180602.

[6] Zuckerman DM, Woolf TB (2004) Systematic Finite-Sampling Inaccuracy in Free Energy Differences and Other Nonlinear Quantities. Journal of Statistical Physics 114: 1303-1323.

### Appendix figure legends

Figure A1. External work distributions for the three stochastic perturbation protocols. a) Normal pulling; b) Constant variance noise *m* = 130 (Chi-square distribution); c) Constant variance noise *m* = 190 (Gaussian distribution; d) AM noise, *m* = 130 (Chi-square distribution); e) AM noise, *m* = 190 (Gaussian distribution); f) FM noise, *m* = 130 (Chi-square distribution); g) FM noise, *m* = 190 (Gaussian distribution). The additional noise reduces the mean value of the external work by helping the polymer to cross the free energy barrier.

Figure A2. External noise amplitudes required for the Jarzynski averaging and the second-order cumulant-expansion formula as functions of the mean work bias (Eqs. 13 and 15).

Figure A3. Two modulation protocols. a) Amplitude Modulation (AM) spring protocol continuously adapts the external noise amplitude to the bias based on the initial, constant-velocity pulling estimate, using the *V*_spring_ function.

b) Frequency modulation (FM) noise protocol randomly applies the external noise with a constant variance according to the probability defined by the *V*_spring_ function.

Figure A4. Cumulant expansion based PMF estimates. a) Estimates based on the stochastic perturbation protocol that uses the Gaussian noise. The noise was applied with the 10 m/s pulling velocity, using four noise amplitudes, 180, 190, 200 and 210.

b) Estimates based on the AM protocol. The Gaussian noise was applied with the 10 m/s pulling velocity using 4 different noise amplitudes, 180, 190, 200 and 210.

c) Estimates based on the FM protocol. The Gaussian noise was applied with the 10 m/s pulling velocity using 4 different noise amplitudes, 180, 190, 200 and 210.

The estimates based on the normal pulling (1 m/s and 10 m/s) are given for the comparison. In each case depicted the computational cost is the same, i.e., it is analogous to the cost required to generate 10,000 trajectories using the normal pulling and 10 m/s velocity.
